# The congenital malformation: Dandy-Walker syndrome (a rare clinical image)

**DOI:** 10.11604/pamj.2026.53.115.49766

**Published:** 2026-03-09

**Authors:** Switi Besekar, Bibin Kurian

**Affiliations:** 1 Department of Child Health Nursing, Smt. Radhikabai Meghe Memorial College of Nursing, Datta Meghe Institute of Higher Education and Research, Wardha, Maharashtra, India

**Keywords:** Ventriculoperitoneal shunt, hypoplasia, cerebellar vermis

## Image in medicine

An 8-month-old male child was admitted with complaints of progressive enlargement of the head, delayed motor responses, lack of coordination, and abnormal eye movements for the past 1 to one and a half months. These symptoms were associated with fever, cough, cold, and recurrent infections. The child had received all age-appropriate immunizations. On physical examination, the child presented with macrocephaly, hypotonia, a bulging anterior fontanelle, lethargy, and irritability, along with characteristic sunsetting eyes and nystagmus. These clinical findings suggested raised intracranial pressure and neurological involvement. The condition may primarily lead to neurological complications, while secondary complications may involve the cardiovascular system, urogenital system, and limb development. Further diagnostic evaluation with computed tomography scan and magnetic resonance Imaging revealed hypoplasia of the cerebellar vermis, cystic dilatation of the fourth ventricle, enlargement of the posterior fossa, and elevation of the torcula, findings consistent with Dandy-Walker syndrome associated with hydrocephalus. The child was managed with surgical placement of a ventriculoperitoneal (VP) shunt to divert excess cerebrospinal fluid and relieve intracranial pressure. In addition to surgical management, the patient received appropriate medical treatment, including Mannitol, furosemide, phenobarbitone, ceftriaxone, and paracetamol. The supportive therapy involves physiotherapy to improve muscle tone and motor development.

**Figure 1:**
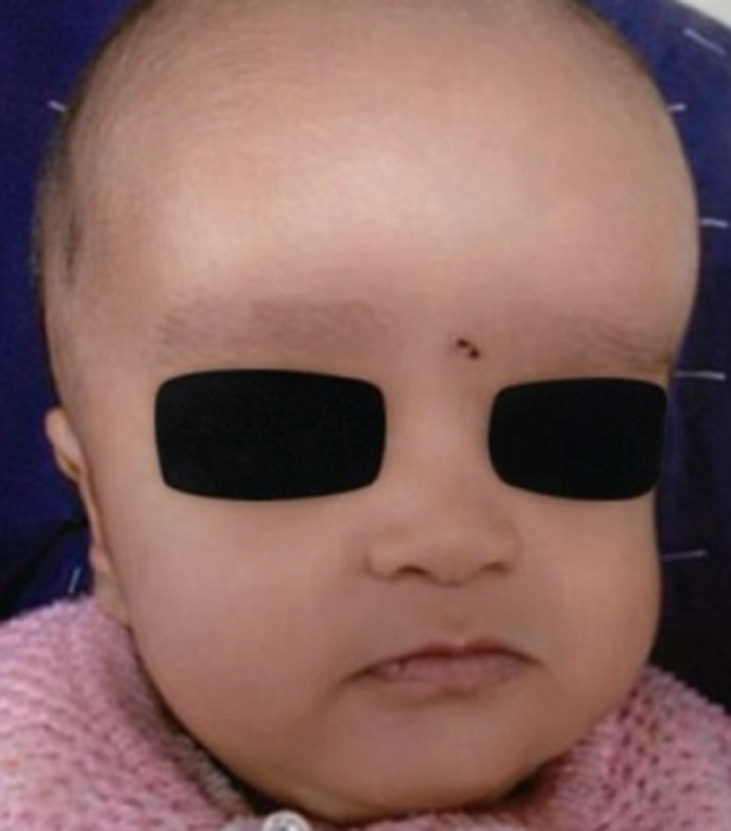
Dandy-Walker syndrome

